# Fall Detection for Shipboard Seafarers Based on Optimized BlazePose and LSTM

**DOI:** 10.3390/s22145449

**Published:** 2022-07-21

**Authors:** Wei Liu, Xu Liu, Yuan Hu, Jie Shi, Xinqiang Chen, Jiansen Zhao, Shengzheng Wang, Qingsong Hu

**Affiliations:** 1 Merchant Marine College, Shanghai Maritime University, Shanghai 201306, China; liu.wei@shmtu.edu.cn (W.L.); liuxuxu99@gmail.com (X.L.); sj1254809611@163.com (J.S.); chenxinqiang@stu.shmtu.edu.cn (X.C.); jszhao@shmtu.edu.cn (J.Z.); szwang@shmtu.edu.cn (S.W.); 2 College of Engineering Science and Technology, Shanghai Ocean University, Shanghai 201306, China; qshu@shou.edu.cn

**Keywords:** BlazePose, long short-term memory neural network, fall detection, deep learning

## Abstract

Aiming to avoid personal injury caused by the failure of timely medical assistance following a fall by seafarer members working on ships, research on the detection of seafarer’s falls and timely warnings to safety officers can reduce the loss and severe consequences of falls to seafarers. To improve the detection accuracy and real-time performance of the seafarer fall detection algorithm, a seafarer fall detection algorithm based on BlazePose–LSTM is proposed. This algorithm can automatically extract the human body key point information from the video image obtained by the vision sensor, analyze its internal data correlation characteristics, and realize the process from RGB camera image processing to seafarer fall detection. This fall detection algorithm extracts the human body key point information through the optimized BlazePose human body key point information extraction network. In this section, a new method for human bounding-box acquisition is proposed. In this study, a head detector based on the Vitruvian theory was used to replace the pre-trained SSD body detector in the BlazePose preheating module. Simultaneously, an offset vector is proposed to update the bounding box obtained. This method can reduce the frequency of repeated use of the head detection module. The algorithm then uses the long short-term memory neural network to detect seafarer falls. After extracting fall and related behavior data from the URFall public data set and FDD public data set to enrich the self-made data set, the experimental results show that the algorithm can achieve 100% accuracy and 98.5% specificity for the seafarer’s falling behavior, indicating that the algorithm has reasonable practicability and strong generalization ability. The detection frame rate can reach 29 fps on a CPU, which can meet the effect of real-time detection. The proposed method can be deployed on common vision sensors.

## 1. Introduction

With the deepening of generalized cognition of the “blue belief” of marine power proposed in the report of the 19th National Congress of the Communist Party of China, the issue of personnel safety in maritime traffic has attracted increasingly extensive attention. According to the “2021 Annual Overview of Marine Casualties and Incidents,” published by the European Maritime Agency (EMSA) [1], from 2014–2020 a total of 550 people went missing in accidents, and 367 people were identified as dead; 89.1% of the victims were seafarers. All but 9.8% of the casualties were due to falling into the water, whereas the rest were due to human slips, trips, and falls. A fall is a sudden, involuntary, unintentional change in body position, falling to the ground or a lower surface, but it does not include behavior resulting from a violent blow, loss of consciousness, stroke, or seizure [2]. Falls can cause non-fatal and fatal injuries to seafarers. Seafarer falls can lead to minor injuries, such as sprains and abrasions. If a fall occurs under special operating conditions, it will also lead to severe injuries, such as fractures and cerebral hemorrhage, which will affect the navigation status of the ship and increase the economic burden on the corresponding family. It also affects seafarers’ quality of life. In order to reduce the injuries and consequences caused by the seafarer’s fall, it is becoming increasingly important to accurately detect the fall of the seafarer onboard and treat the fallen seafarer in a timely manner. Therefore, research on detecting seafarers’ falls has very significant social importance.

The seafarer fall detection algorithm, based on common vision sensors, such as an RGB camera, refers to the process of judging whether a seafarer member falls, after collecting continuous video image frames through RGB camera equipment for intelligent image processing. The main process is as follows: First, preprocess the original video image (such as box plot [3] data cleaning), and then extract features from the preprocessed data, and finally use a pre-trained classification algorithm (such as long short-term memory neural network [4,5]) to perform fall detection based on the features. 

Because the human body key point information extraction algorithms now commonly used, such as OpenPose [6], AlphaPose [7], and other networks, are easily affected by factors such as occlusion caused by seafarer operations, many important features that can represent the current state of the seafarer will be lost. Therefore, this study uses the BlazePose human body key point information extraction network [8] to extract feature information. Although BlazePose can solve the occlusion problem very well, when the network restarts the pre-trained SSD body detection model to obtain the human bounding box, it causes the loss of human key point information. To address this issue, we propose a method that uses an offset vector to update the human body bounding box obtained by the optimized head detector, which greatly guarantees the stability of network extraction of key point information and reduces the weight of the network to a certain extent, making it more suitable for layouts on mobile devices and ordinary vision sensors. 

The judgment of fall behavior not only requires the data of the fall but also relies heavily on the data before the fall. Based on this, this paper presents a fall detection algorithm based on a long short-term memory neural network that is suitable for dealing with long-term dependency problems. This network can avoid gradient disappearance and gradient explosion problems caused by ordinary recurrent neural networks. Experiments show that the BlazePose–LSTM seafarer fall detection model proposed in this paper can be better applied to the seafarers’ working environment and can be arranged on general computing power equipment.

The main contributions of this paper are as follows: (1) A new seafarer fall detection model based on optimized BlazePose–LSTM is proposed. Fall discrimination depends on a long sequence; therefore, LSTM was used for fall discrimination. Owing to its special gating unit, the long-dependence problem is well resolved. (2) The offset vector is used to update the human body bounding box generated by the optimized head detector, which reduces the need to repeatedly start the head detection model, speeding up the fall detection efficiency of the model, and making it possible to arrange onboard RGB vision sensors with lower computing force. (3) The method proposed in this paper can solve the problem of the inability to perform fall detection owing to occlusion and self-occlusion, and it still has good generalization ability in complex work environments.

## 2. Related Works

Currently, methods for realizing seafarer fall detection can be roughly divided into three types: (1) seafarer fall detection methods based on environmental sensors, (2) seafarer fall detection methods based on wearable sensors, and (3) seafarer fall detection methods based on vision sensors. The seafarer fall detection method based on environmental sensors mainly arranges relevant equipment in the seafarer activity environment to record seafarer activities in the current environment, and then fuses the information collected by environmental sensors, such as pressure sensors and acoustic sensors [9], to detect seafarer falls. However, this method is easily affected by the environment, requires more information monitoring equipment, and can only detect seafarer falls in fixed areas [10], such as indoors. Wearable sensor-based seafarer fall detection methods mainly use specific wearable MEMS devices [11,12,13], such as belts and smart watches, to monitor various physical signs of seafarers. If there was an abnormality in various indicators of the body, and the abnormality was consistent with the characteristics of a fall, it was judged as a fall. For example, Desai et al. [14] designed a belt using a simple 32-bit micro-controller. This belt can not only detect falls for the first time, but also send distress information to the family members of the fallen person via the GSM module. In addition, it can also be equipped with real-time safety mechanism gear to minimize injury in the case of a fall. However, most seafarer members wear safety helmets, safety ropes, and life jackets while working on ships. If one continues to wear other wearable devices, not only will one obtain inaccurate data owing to the influence of the work equipment, but it will also affect the seafarer’s work.

With the development of vision algorithms, an increasing number of new lightweight classification networks have been deployed on vision sensors, and vision tasks can be achieved without relying on computing power. The video image data acquired by common vision sensors, such as RGB cameras, are then used for seafarer fall detection using the trained classification model. For example, H. Abdo et al. [15] used RetinaNet to detect people in videos and obtain motion features and human shape features (including the aspect ratio of the human bounding box and motion history images), and then input the improved mobile nets to determine whether they fell or not; Y. Chen et al. [16] used an OpenPose–SVM combination algorithm to detect people falling, which can accurately determine whether people tend to fall. The experiments proved that they achieved recognition rates of 92.5% and 95.8% on the two public data sets of MCFD and URFD, respectively; Ramirez et al. [17] used the AlphaPose–kNN combination algorithm to detect people falling. First, AlphaPose was used to obtain the skeleton information of the human body; the information was then input into the kNN network for classification. An accuracy rate of 99.51% was obtained on the public UP-Fall dataset.

Among seafarer fall detection methods based on vision sensors, commonly used fall detection algorithms are based on the threshold method [18] and machine learning [19]. The threshold method mostly extracts human body features that can characterize human motion information, preprocesses them with statistical methods, and then compares them with a preset threshold, where the threshold is more dependent on the extracted motion information. The fall detection algorithm, centered on the machine learning algorithm, converts the seafarer’s fall behavior into a multi-classification problem to classify the falling behavior and other similar behaviors. The threshold method extracts features from the preprocessed data, and uses machine learning algorithms to build a seafarer detection model. Common machine learning algorithms include random forests [20], artificial neural networks [21], and decision trees [22]. For example, Younis et al. [23] used a support vector machine as the core and a novel feature discriminatory trait of fall and non-fall events. These features were then used to train the support vector machine for classification. Experiments show that the proposed method is highly effective for detecting falls. However, these two methods are highly dependent on the features extracted by the experimenter and are subjective and arbitrary. Therefore, the feature selection will be biased, and the movement state of the seafarer cannot be well represented. The deep learning method can be used to extract fall features to reduce the experimental bias caused by the experimenter. Many studies have focused on deep learning algorithms to realize the fall detection of seafarers. Commonly used deep learning methods include convolutional neural networks [24], recurrent neural networks [25], and long short-term memory neural networks [26]. For example, Maitre et al. [27] proposed a fall behavior recognition algorithm based on a hybrid CNN and LSTM model. The model adopts a two-layer structure. The radar data were fed into the network in groups of 15, with 95% overlap between each group of data. The CNN extracts the spatial features of the video sequence. The LSTM extracts the features in the temporal dimension of the data, and finally uses the SoftMax classifier for recognition. Experiments showed that this method could effectively improve the accuracy of fall recognition, reaching a recognition accuracy of 98.5%.

## 3. Fall Detection Algorithm for Shipboard Seafarers Based on BlazePose and LSTM 

### 3.1. Human Body Key Point Extraction Network Based on BlazePose

Existing human key point extraction networks, such as OpenPose, AlphaPose, and OpenPifPaf [28], are extremely vulnerable to dense crowd occlusion or human self-occlusion, resulting in the low performance of human key point detectors.

**Figure 1:**
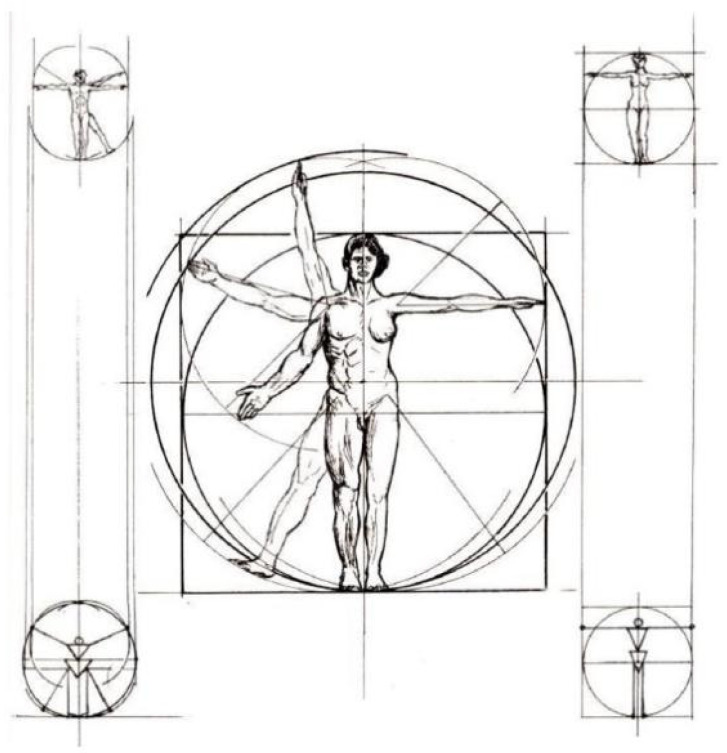
Vitruvian theory of the human body.

In order to solve this problem, this study selects the BlazePose human body key point extraction network to extract human body key point information. BlazePose is a lightweight convolutional neural network. Unlike most convolutional neural networks, NMS cannot be used for post-processing because human behaviors, such as waving and walking, have a large degree of freedom. Inspired by the Vitruvian theory [29], in the process of obtaining the key points of the entire human body in BlazePose, its warmup module first obtains the shoulder center and hip center of the human body through the pre-trained SSD body detection model, and uses this to predict the remainder of the human body topology key points. According to further research on the Vitruvian theory, it was found that the ratio of the human head to the entire human body is close to 1:7.5 in Figure 1. In order to cope with the particularities of different people, this study used a ratio of 1:8 to represent the entire human body, with the human head, so that a head detector can be used to replace the pre-trained SSD body detector.

In the process of the key point information regression of the optimized BlazePose human key point network, we will obtain the head bounding-box information ($f_{x}$, $f_{y}$, $f_{w}$, $f_{h}$) and the coordinates of the midpoint of the hip ($h_{x}$, $h_{y}$). According to the Vitruvian body theory, we can obtain the bounding-box information of the human body ($b_{x}$, $b_{y}$, $b_{w}$, $b_{h}$) and its rotation angle. The derivation formula is as follows:

(1) $$b_{x}=f_{x}+0.5\times f_{w}-4\times f_{h}$$

(2) $$b_{y}=f_{y}$$

(3) $$b_{w}=8\times f_{h}$$

(4) $$b_{h}=8\times f_{h}$$

(5) $$\theta =\arctan\frac{h_{x}-(f_{x}+0.5\times f_{w})}{h_{y}-(f_{y}+0.5\times f_{h})}$$

**Figure 2:**
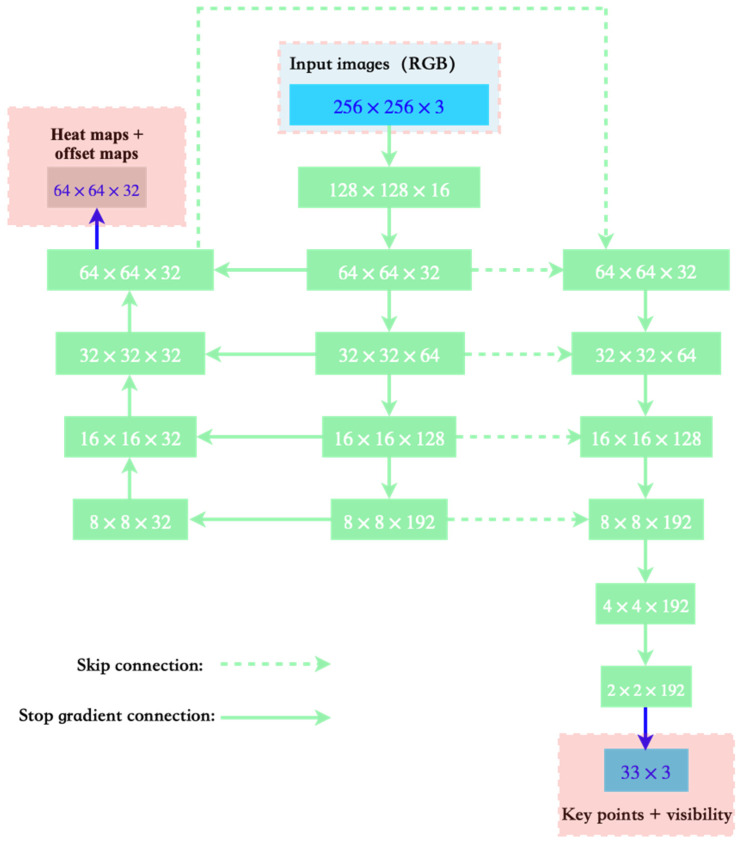
The network structure of BlazePose.

The training process of the BlazePose network is mainly divided into two parts: key point detection and key point regression. During the training process of the network, the entire network adopted a combination of heat map, offset, and regression, corresponding to the heat map in Figure 2 (left), offset in Figure 2 (middle), and regressing in Figure 2 (right).

First, the network used heatmaps and offsets for training. However, during the regression training, the network clipped the output branch shown in Figure 2 (left). This not only effectively uses the heatmap to supervise the training process, but also improves the inference speed of the entire network without losing accuracy.

**Figure 3:**
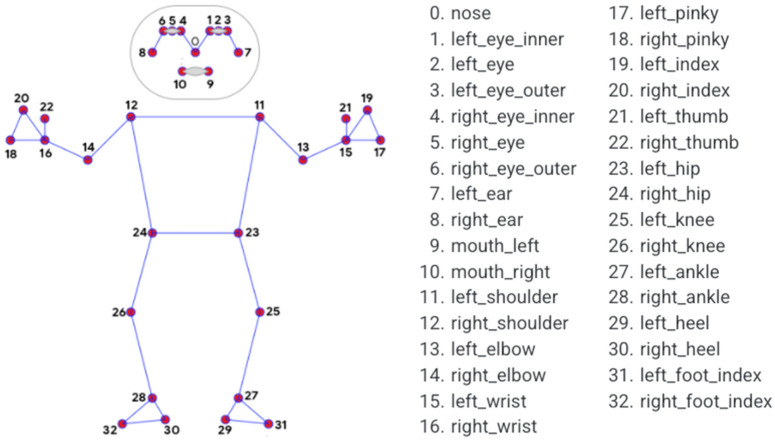
Human body key point topology map of BlazePose.

Compared with the 17 human key points of the COCO Pose, the BlazePose human key point extraction network predicted 33 human key points (including confidence) for each human body. The topology of the human key points is shown in Figure 3. Owing to the richer semantic features obtained, the accuracy of fall detection in this study was also guaranteed.

**Figure 4:**
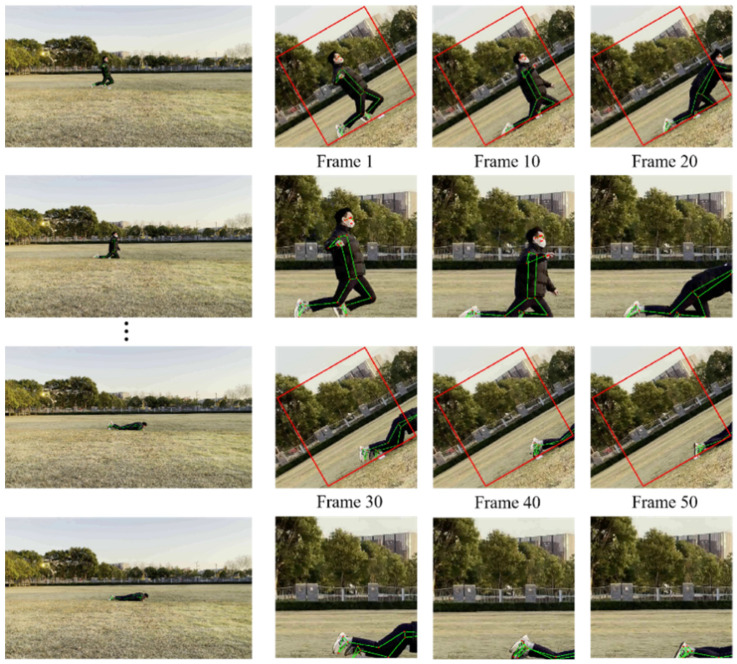
The detection process of BlazePose human key point information extraction network.

In order to arrange the human fall detection algorithm designed in this study on ordinary visual sensors, this study optimizes the preheating module of BlazePose and uses the offset vector to update the bounding box of the moving human body, thereby reducing the detection frequency of the human body, and improving the speed of human key point information detection. Figure 4 shows the image processing using the traditional BlazePose human body key point extraction network. First, after the video stream is input to the network, the pre-trained SSD body detector preset in the network will obtain the human body bounding box in the first frame image, as shown in Figure 4 (top), in the first frame. At this point, we can clearly observe the human key point information extracted by the BlazePose network in the current frame image with the naked eye, and this bounding box will be used in subsequent frame images. In this case, we observed the next 50 frames of images, and exported the image rendering results every 10 frames. In the process, we observed that after the video stream was continuously input to the network, the first frame of the current segment was always used for human detection and subsequent human key point information. We found that after more than 20 frames, the human body within the bounding box begins to become mutilated. In order for BlazePose to efficiently obtain the key points of the human body, it primarily needs to determine the shoulder center point and the hip center point, but the human body in the bounding box has begun to no longer satisfy these conditions. Although BlazePose has superior prediction ability, the network can still predict the key point information of the incomplete part of the human body, but the quality of the key point information is not satisfactory. The cropped rendering of the bounding box in Figure 4 (bottom) clearly shows that a fall occurred when this video clip was input into the network for more than 20 frames. However, based on the information obtained, it was difficult to complete the fall assessment in the current state. After this happens to the network, the network restarts the pre-trained SSD body detection module to obtain a new human body bounding box. However, because the semantic information in the previous bounding box is lost, it can easily cause misjudgment.

### 3.2. Optimized Bounding-Box Detector Based on Offset Vector

**Figure 5:**
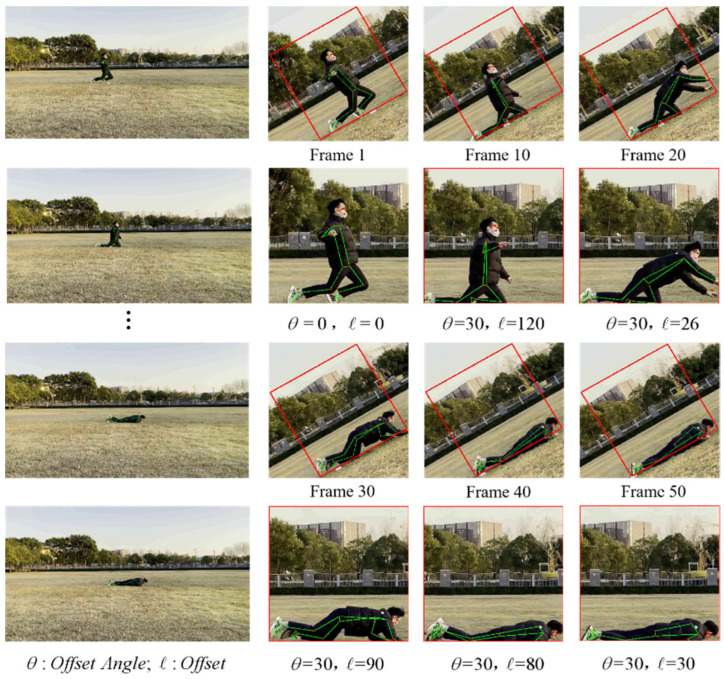
The network detection process of the optimized human body key point information extraction based on BlazePose.

To solve the above-mentioned defects of the BlazePose network and reduce the dependence of the network on computing power, this study adopts the method of updating the bounding box with the offset vector. This method ensures that the human body obtained by the first frame processing is always maintained in the center of the entire bounding box. This process is illustrated in Figure 5.

In this study, the images were still taken every 10 frames for comparison. We observed that the position of the bounding box was updated using the offset vector. During these 50 frames, the head detection module was not restarted to obtain the human bounding box, and the person in the image was always kept in the middle of the image. After the optimization of the network, the use of rigid feature detectors is reduced, which greatly reduces the dependence of the human body on key point information extraction based on the BlazePose network on computing power. Simultaneously, owing to the reduction in the activation of the head detector, the speed of the key point information extraction of each frame of the image is improved by 0.04 s.

**Figure 6:**
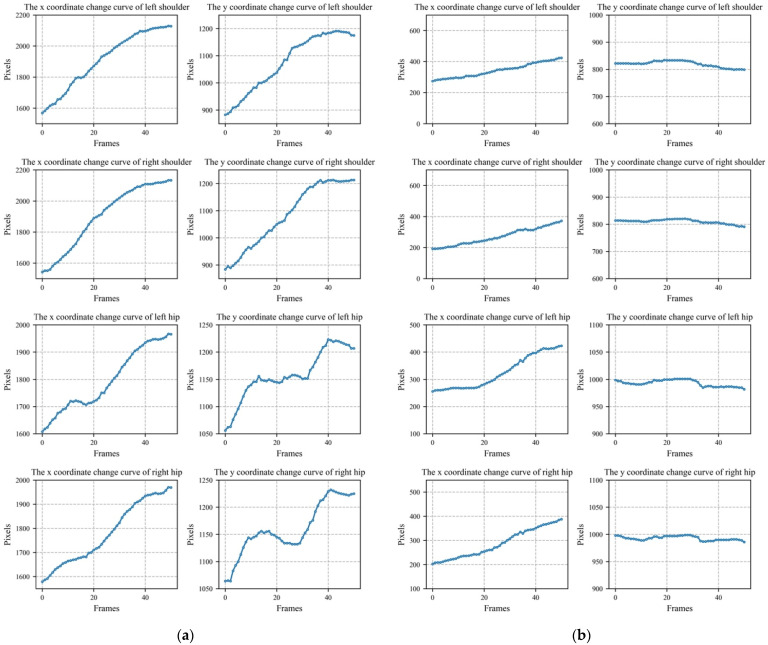
The changed curve of the human body key point information. (**a**) Falling. (**b**) Walking.

Through the optimized human body key point information extraction based on BlazePose, we can obtain more stable key point information. From this information, we selected four key points with relatively low degrees of freedom, namely the left shoulder, right shoulder, left hip, and right hip. As the time sequence changed, the information change curves of the four key points are shown in Figure 6. The abscissa of each subgraph is the image in time series, and its unit is the frame. Combined with the title of the picture, the ordinate is the x or y pixel position of the key point information of the human body.

By cleaning the data using the boxplot method, abnormal data, such as missing key point information due to restarting the head detector, are optimized. According to the Panax notoginseng criterion of deep learning, it can be guaranteed that a classification model with better generalization ability can be obtained after feeding into the long short-term memory neural network for seafarer behavior classification training.

### 3.3. Long Short-Term Memory Neural Networks

Falls and other human behaviors can often be viewed as continuous sequences, whose spatial and temporal characteristics are very important. Recurrent neural networks (RNN) [30,31] are particularly powerful for processing time-series data because of their excellent memory. To solve the problem of gradient explosion and disappearance of RNN to process long sequence data, this study uses a long short-term memory neural network (LSTM) [32] to judge whether a person produces falling behavior. The LSTM is a special RNN that can effectively solve long-term dependence problems. This study used a self-made gesture dataset (4 K, 60 frames), and inputted the LSTM and RNN networks for training. The results are presented in Table 1.

As shown in the table, the validation accuracies of the two networks for sequence data with more than 120 frames of images are very different, and that of the RNN network is as low as 36%, which obviously does not meet the requirements of this study. In contrast, LSTM can obtain good results in both designed experiments.

**Figure 7:**
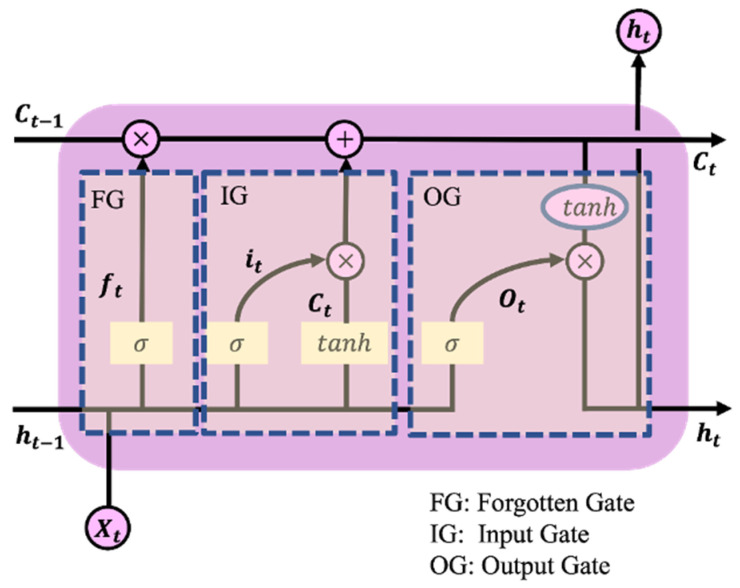
Unit structure diagram of LSTM [34].

The basic unit of LSTM is a memory block mainly formed by a memory cell and three gate control units (including an input gate ($i_{t}$), an output gate ($o_{t}$), and a forget gate ($f_{t}$)) [33]. The internal structure of each unit is shown in Figure 7. The memory unit is represented by the horizontal straight line at the top of Figure 7, which is used to receive information from the previous moment and transfer the processed information to the next moment.

For each LSTM cell, the first step is to decide which information from the previous LSTM unit needs to be forgotten. This decision was made by the forget gate. The gating unit reads $h_{t-1}$ and $x_{t}$, and then outputs a value $f_{t}$ between 0 and 1 to update the state $C_{t-1}$ of the current LSTM unit. In the process of fall detection, the forget gate selectively stores and forgets the information transmitted by the previous LSTM unit, and the information transmitted by the input gate. Then, the forget gate stores the series of frame images that are determined to fall in behavior recognition. The calculation formula is as follows:

(6) $$f_{t}=\sigma (U_{t}h_{t-1}+W_{f}x_{t})$$

(7) $$k_{t}=c_{t-1}⊙f_{t}$$

where $x_{t}$ is the human body key point information group passed by the input gate, $h_{t-1}$ is the output of the previous LSTM cell, σ is the sigmoid layer, $U_{t}$ is the input coefficient matrix of the forget gate, $W_{f}$ is the network coefficient matrix of forget gate, $c_{t-1}$ is the cell state of the previous LSTM cell, and $k_{t}$ is the output of the forget gate, which is used to update the current cell state.

After forgetting the current state information of the LSTM unit, the input gate processed the information input into the current unit. First, it is necessary to use $h_{t-1}$ and $x_{t}$ to combine with the sigmoid layer to determine the information that needs to be updated in the current unit. Second, the activation function tanh is used to process $h_{t-1}$ and $x_{t}$ to obtain new candidate unit information as supplementary information. The calculation formula is as follows:

(8) $$i_{t}=\sigma (U_{i}h_{t-1}+W_{i}x_{t})$$

(9) $$g_{t}=tanh(U_{g}h_{t-1}+W_{g}x_{t})$$

(10) $$j_{t}=g_{t}⊙i_{t}$$

(11) $$c_{t}=j_{t}+k_{t}$$

where $i_{t}$ is the output information after parallelization; $g_{t}$ is the candidate input information; $U_{i}$ is the input coefficient matrix of input gate; $W_{i}$ is the network coefficient weight matrix of the input gate; $U_{g}$ is the candidate input information weight coefficient matrix; $W_{g}$ is the weight coefficient matrix of the input gate; $j_{t}$ is the output of the input gate, which will used to update the current cell state; and $c_{t}$ is the updated unit status.

Finally, the current LSTM unit determines the output value. First, the sigmoid layer is used to obtain the output judgment condition, and then a layer tanh is used to obtain an inter-decision vector in [−1, 1]. This vector was multiplied by the result obtained from the input gate to obtain the final LSTM unit output value. In the process of fall detection, the output gate normalizes the final information to classify falls and other actions. The specific formula is as follows:

(12) $$o_{t}=\sigma (U_{o}h_{t-1}+W_{o}x_{t})$$

(13) $$h_{t}=tanh(c_{t})⊙o_{t}$$

where $o_{t}$ is the output information after parallelization, $U_{o}$ is the input coefficient matrix of the output gate, $W_{o}$ is the network coefficient weight matrix of the output gate, and $h_{t}$ is the output information of the current LSTM cell.

Therefore, LSTM has unique advantages in processing long-sequence data compared with RNN. The experiments conducted in this study achieved sufficiently good results.

## 4. Dataset and Experimental Analysis

### 4.1. Experimental Dataset

When seafarers work on ships, their behaviors are not as complicated as those on land, and activities such as crouching down and lying down are rare. Therefore, the main human activities in the videos captured in this study were walking, standing, sitting, and falling. This study constructed a self-developed dataset based on these four activities. This study selected two male samples and one female sample. Of the three samples, two were younger and their data were taken in the field, and the other was from a seafarer whose data was taken in the cabin. The specific information is shown in Table 2. The body types of the three people were different. It is representative. 

To enrich the datasets, we extracted all the videos about these four kinds of activities from the URFall public dataset and the FDD public dataset. Then, we processed all the videos. We extracted and labeled the human body key point information for each frame image. Finally, 11,292 groups were obtained, of which 3370 groups were from our own videos, 2995 groups were from the URFall public dataset, and 4527 groups were from the FDD public dataset. The detailed distribution of the dataset is presented in Table 3:

Finally, we divided all the obtained data into training dataset, validation dataset, and test dataset, at a ratio of 7:2:1. The training set accounted for 70% of the total dataset, and approximately 7904 sets of key point information. The validation set accounted for 20% of the total data, approximately 2258 sets of key point information, and the remaining 10% contained about 1129 sets of key point information as the test set.

### 4.2. Experimental Environment

Table 4 lists the experimental conditions of the data training completed in this study. 

### 4.3. Experimental Results

**Figure 8:**
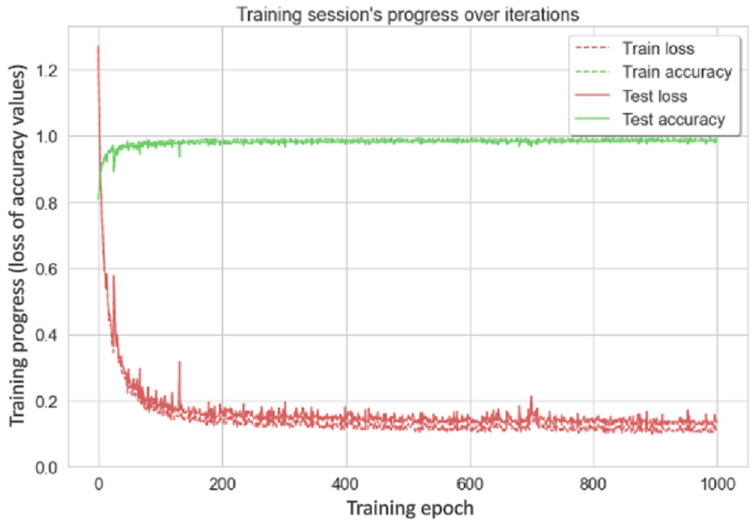
Iterative process of model loss.

**Figure 9:**
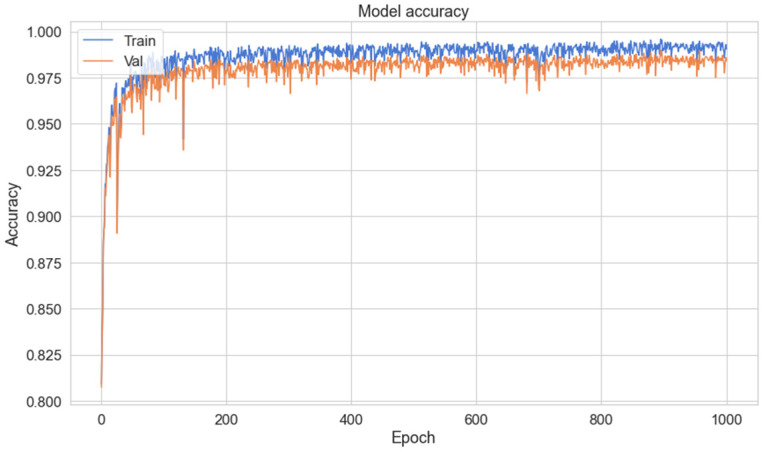
Iterative process of model accuracy.

To make the seafarer fall detection algorithm designed in this study efficiently applicable in a real shipboard operating environment, the dataset used in this study contains the daily activities of walking, falling, sitting, and standing associated with falls. The Adam optimization method was used to optimize the network, and the ReLU method was used between the network layers to improve the generalization ability of the model. Finally, the fully connected layer and the SoftMax classifier were used to calculate the final classification results. Figure 8 and Figure 9 show the variation curves of the loss and accuracy of the training and validation sets with the iteration process during the training process.

According to the change in the curve, it is appropriate to use the left shoulder, right shoulder, left hip, and right hip to describe the movement state of the personnel.

**Figure 10:**
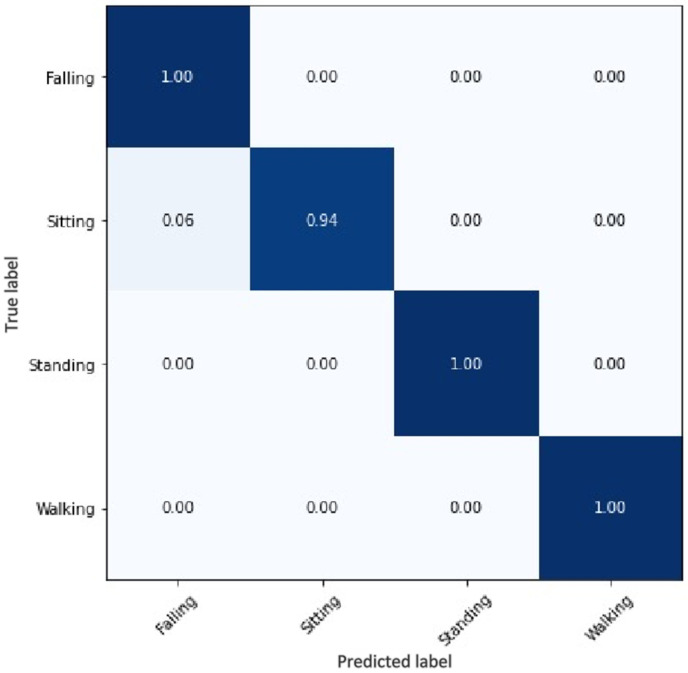
Confusion matrix of training results.

**Figure 11:**
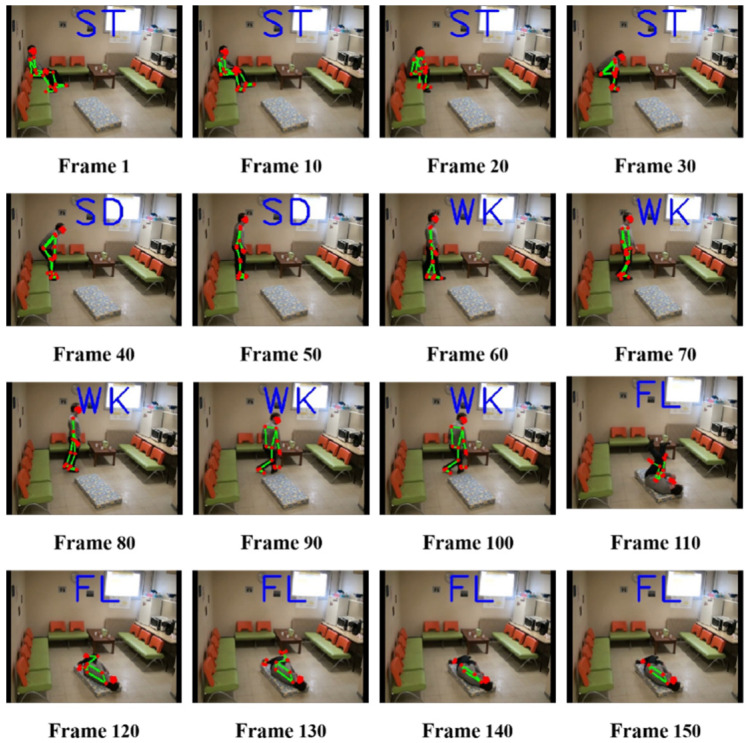
Detection results. (Note: ST, sitting; SD, standing; WK, walking; FL, falling).

Because accidental falls are a significant threat to personal safety in various work scenarios, this study allows other behaviors to be falsely detected as falls. This means that specificity is less than 1, but the accuracy and sensitivity indicators must be as optimal as possible. A fuzzy matrix was used in this study to analyze the final results. The results are shown in Figure 10:

As shown in the figure above, the human fall detection algorithm proposed in this study can effectively distinguish the fall situation from the daily situation. The sitting behavior is similar to the falling behavior, and misjudgment occurs in it, but the desired effect of the algorithm designed in this study has been obtained well. Moreover, the optimized human body key point information extraction, based on the BlazePose network, in this study can reach a frame rate of 25+ FPS, provided by Google Labs on the experimental equipment described in this study, without using a GPU. This can be effectively placed on mobile devices, as well as on common RGB cameras for real-time detection and cost savings. We rendered some of the running results using the video dataset, and the detection results are shown in Figure 11.

Based on the BlazePose–LSTM seafarer fall detection model, the accuracy rate of seafarer falls in this study reached 100%, the recognition rate for non-falls reached 97.95%, and the average detection frame rate reached 29 frames/s. It can be proved that the BlazePose–LSTM network model proposed in this study has high recognition accuracy and a fast frame rate for the experimental data set. Table 5 shows a performance comparison between the model in this study and the methods used in other studies, which further shows that this method exhibits a certain degree of improvement in accuracy, specificity, and sensitivity [35]. The calculation formulae for the three indicators are as follows:

(14) $$Accuracy=\frac{TP+TN}{TP+TN+FP+FN}\times 100\%$$

(15) Sensitivity=TP/(TP+FN)×100%

(16) Specifity=TN/(TN+FP)×100%

According to the statistics and comparison information in the above table, the BlazePose–LSTM seafarer fall detection network structure proposed in this study increased the accuracy, specificity, and sensitivity by 4.57%, 1.7%, and 9%, respectively, compared with the OpenPose-YOLO [36] fall detection network. The fall recognition rate for seafarers increased by nearly five percentage points, and the false alarm rate was much lower. Compared with CNN [37], the performance also exhibits a certain improvement. Compared with the stacked LSTM [38], the accuracy, specificity, and sensitivity increased by 3.06%, 2.06%, and 3.66%, respectively.

In summary, the seafarer fall detection model based on BlazePose–LSTM proposed in this study can obtain better detection accuracy and frame rate. In addition, for the fall detection of seafarer members on ships, the purpose of using the algorithm is to detect all fall behaviors so that medical rescue can be performed in time, to prevent irreversible physical damage to the seafarer members. Therefore, it is possible to have a fall-like behavior and be misjudged as a fall, but it is impossible to miss the detection of a fall behavior; therefore, the accuracy and sensitivity indicators are also very important.

### 4.4. Generalization Experiment of Seafarer Fall Detection on Ships Underway

**Figure 12:**
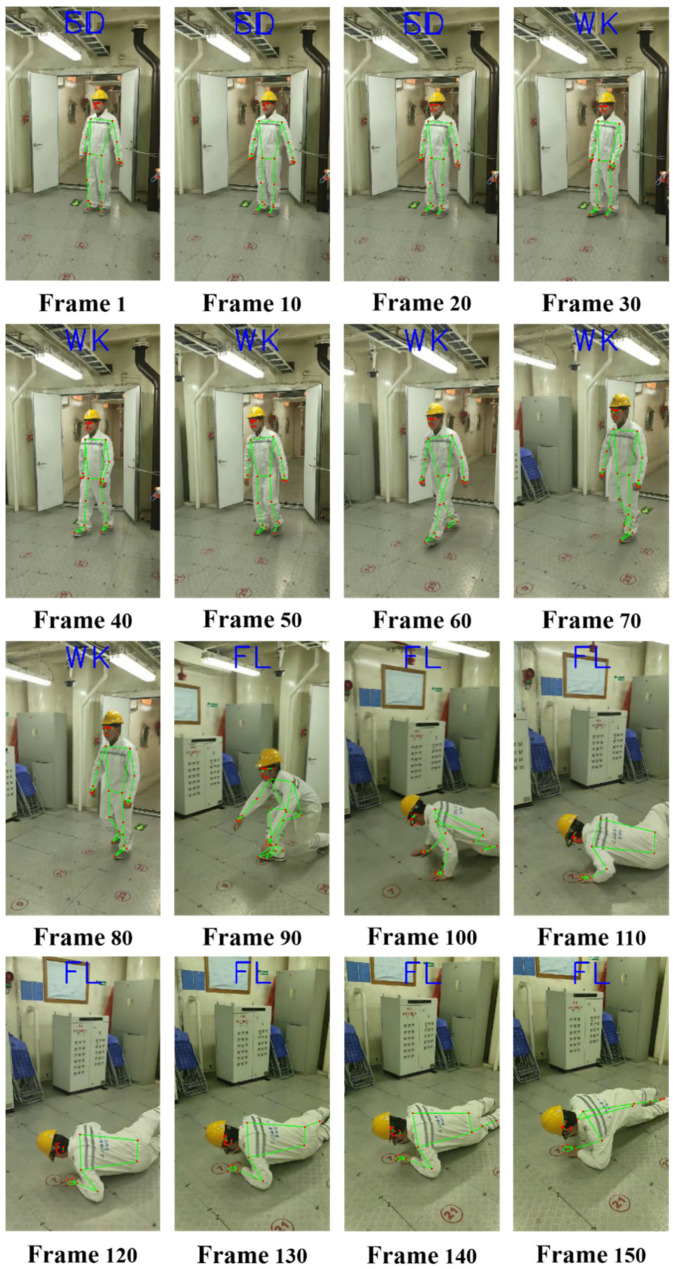
Detection results.

Note that our experiments were carried out under the condition that the ship’s sailing state is relatively stable. In order to verify the generalization ability of the algorithm in this study in an actual ship environment, we collected videos of several volunteers simulating ship operations on the *Wu Song* cargo ship. All the videos were captured using onboard RGB cameras. The detection results for one of the video sequences are shown in Figure 12:

In this video sequence, it can be detected that the current image of the seafarer member is “SD,” which is the standing state label in the first 30 frames. When the seafarer starts to walk, the algorithm can quickly respond and output “WK,” which is the walking label. When the detection reaches about the 90th frame, the algorithm detects that the current seafarer member has demonstrated the features of a fall, and quickly outputs the “FL” label, which shows that the seafarer member in the current image has fallen. The above analysis shows that the BlazePose–LSTM seafarer fall detection algorithm proposed in this study has a good generalization ability in actual ship seafarer fall detection.

## 5. Conclusions

Based on the optimized BlazePose–LSTM seafarer fall detection model, this study first uses the human body key point information extraction based on the optimized BlazePose network to detect the head and obtain the human body feature information. In this study, according to the Vitruvian theory, a head detector was designed to replace the pre-trained SSD body detector. After obtaining the human bounding box, an offset vector is designed to update the human body bounding box, so that the head detector built into the network does not need to be repeatedly enabled. Subsequently, the extracted information is cleaned by the box plot, and then the LSTM is used to determine whether the seafarer has fallen. At the same time, this study verifies the method proposed in this paper on the URFall public data set and the FDD public data set, which proves that the method can obtain a high accuracy and detection frame rate. In the future, we will evaluate the performance of our method in the case of multiple falls. In addition, the algorithm can be arranged on a mobile device to realize industrial applications.

## Figures and Tables

**Table 1:** Comparison of LSTM and RNN.

Network	Comparison		
	Accuracy	Verify Image	Image Resolution
LSTM	89%	500	1920 × 1080
RNN	36%	500	1920 × 1080
LSTM	97%	100	720 × 480
RNN	91%	100	720 × 480

**Table 2:** Self-developed dataset construction.

Sample	Age	Height	Weight	Sex	Environment
Sample 1	24	168 cm	62 Kg	Male	Field
Sample 2	25	162 cm	47 Kg	Female	Field
Sample 3	39	176 cm	74 Kg	Male	Cabin

**Table 3:** Dataset source statistics.

Dataset Source	Data Quantity	Data Proportion	Data Acquisition Equipment
Self-made dataset	3770	33.38%	RGB Camera
URFall public dataset	2995	26.52%	Kinect Camera
FDD public dataset	4527	40.09%	RGB Camera

**Table 4:** Experimental hardware configuration.

Experimental Conditions	Parameters
CPU	Intel(R) Xeon(R) CPU E5-1650 v2 @ 3.50 GHz 3.50 GHz
GPU	GeForce GTX970
Memory	8 G
Hard disk	1 T
System	Windows 10 Professional Edition
Language	python3.8
Frame	TensorFlow1.15.5
Software	Jupyter Notebook

**Table 5:** Performance comparison between BlazePose–LSTM seafarer fall detection model and other models.

Models	Accuracy	Specificity
OpenPose-YOLO	95.43%	96.8%
CNN	96.97%	95.44%
Stacked LSTM	96.94%	97.15%
BlazePose–LSTM (Ours)	100%	98.5%

## Data Availability

Not applicable.
